# Sprint-based exercise and cognitive function in adolescents

**DOI:** 10.1016/j.pmedr.2016.06.004

**Published:** 2016-06-07

**Authors:** Simon B. Cooper, Stephan Bandelow, Maria L. Nute, Karah J. Dring, Rebecca L. Stannard, John G. Morris, Mary E. Nevill

**Affiliations:** a Sport, Health and Performance Enhancement (SHAPE) Research Centre, Department of Sport Science, School of Science and Technology, Nottingham Trent University, Nottingham, UK; b School of Sport, Exercise and Health Sciences, Loughborough University, UK

**Keywords:** High-intensity exercise, Executive function, Memory, Information processing

## Abstract

Moderate intensity exercise has been shown to enhance cognition in an adolescent population, yet the effect of high-intensity sprint-based exercise remains unknown and was therefore examined in the present study. Following ethical approval and familiarisation, 44 adolescents (12.6 ± 0.6 y) completed an exercise (E) and resting (R) trial in a counter-balanced, randomised crossover design. The exercise trial comprised of 10 × 10 s running sprints, interspersed by 50 s active recovery (walking). A battery of cognitive function tests (Stroop, Digit Symbol Substitution (DSST) and Corsi blocks tests) were completed 30 min pre-exercise, immediately post-exercise and 45 min post-exercise. Data were analysed using mixed effect models with repeated measures. Response times on the simple level of the Stroop test were significantly quicker 45 min following sprint-based exercise (R: 818 ± 33 ms, E: 772 ± 26 ms; *p* = 0.027) and response times on the complex level of the Stroop test were quicker immediately following the sprint-based exercise (R: 1095 ± 36 ms, E: 1043 ± 37 ms; *p* = 0.038), while accuracy was maintained. Sprint-based exercise had no immediate or delayed effects on the number of items recalled on the Corsi blocks test (*p* = 0.289) or substitutions made during the DSST (*p* = 0.689). The effect of high intensity sprint-based exercise on adolescents' cognitive function was dependant on the component of cognitive function examined. Executive function was enhanced following exercise, demonstrated by improved response times on the Stroop test, whilst visuo-spatial memory and general psycho-motor speed were unaffected. These data support the inclusion of high-intensity sprint-based exercise for adolescents during the school day to enhance cognition.

## Highlights

• First study to examine the effects of high intensity, sprint-based exercise
• Multiple components of cognitive function and time course of the effects examined
• Speed of executive function enhanced immediately and 45 min post-exercise
• No effect of exercise on visuo-spatial memory or general psychomotor speed
• Opportunities for high intensity activity should be incorporated in the school day

## 1 Introduction

There is a substantial body of literature suggesting that exercise has beneficial effects upon cognitive function in adult populations (Chang et al., 2012, Lambourne and Tomporowski, 2010). Although less work has been conducted in adolescents, there is some evidence to suggest that exercise is also beneficial for cognitive function in this population (Best, 2010, Cooper et al., 2012b, Ellemberg and St-Louis-Deschênes, 2010, Hillman et al., 2009, Stroth et al., 2009) where it is of particular importance for academic achievement (Morales et al., 2011, Tomporowski et al., 2015). The viewpoint that exercise is beneficial for cognition in young people is also supported by an effect size of 0.32 in the meta-analysis of Sibley and Etnier (2003).

However, the literature surrounding the effects of exercise on cognitive function can be difficult to interpret due to the many moderating variables in the exercise-cognition relationship, including the intensity, duration and modality of the exercise, as well as the component of cognitive function examined (Chang et al., 2012). Of particular interest in young people is the effect of high intensity intermittent activities, given that this is the mode of exercise that young people most frequently engage in (Armstrong and Welsman, 2006, Bailey et al., 1995, Howe et al., 2010). Specifically, it has been suggested that 95% of young people's ‘bouts’ of physical activity are less than 15 s in duration (Bailey et al., 1995) and evidence has shown that young people's activity patterns are sporadic and very rarely consist of sustained moderate or vigorous intensity activity (Armstrong and Welsman, 2006). Despite this the studies examining the effect of exercise on young people's cognitive function have focussed upon more continuous exercise models such as walking (Hillman et al., 2009), running (Cooper et al., 2012b) and cycling (Ellemberg and St-Louis-Deschênes, 2010), with no studies to date examining the effects of high intensity intermittent exercise.

In adults (27 males), one study has compared the effects of high intensity exercise (2 × 3 min running sprints) and moderate intensity exercise (40 min running) on learning performance (Winter et al., 2007). Interestingly, the findings suggest that high intensity exercise was the most beneficial for immediate learning performance, indicative of enhanced cognitive function (Winter et al., 2007). The authors go on to suggest that these effects may be influenced by higher brain-derived neurotrophic factor (BDNF) and catecholamine concentrations following the high intensity exercise (Winter et al., 2007). However, this study was conducted in an adult population and meta-analyses have suggested that age is a moderating variable in the exercise-cognition relationship (Chang et al., 2012, Sibley and Etnier, 2003), potentially due to the fact that young people have a larger brain weight relative to their body weight and a greater metabolic rate per unit of brain weight (Hoyland et al., 2009).

In addition to the above differences in the BDNF and catecholamine response to high intensity intermittent activity (compared to the more commonly assessed moderate intensity activity), psychological mechanisms such as exercise-induced arousal and mood may also moderate the exercise-cognition relationship. The increase in arousal during and following exercise has been suggested to affect cognitive function (McMorris, 2009), with exercise intensity affecting subsequent changes in arousal (Kamijo et al., 2004). Furthermore, mood has been suggested to affect cognitive function and exercise of differing intensities has been shown to differentially affect mood (Kennedy and Newton, 1997). Therefore, despite clear scientific rationale, the effect of high intensity exercise on cognition in adolescents has not yet been examined.

It is also important that a number of domains of cognitive function are examined due to evidence suggesting that exercise differentially affects different domains of cognitive function (Chang et al., 2012). For example, in the meta-analysis of Chang et al. (2012) exercise was shown to enhance executive function but have no effect on memory. Furthermore, it is also important for studies to examine the time-course of the changes in cognitive function following exercise, with much of the literature to date only examining cognitive function immediately following exercise (Ellemberg and St-Louis-Deschênes, 2010, Stroth et al., 2009). However, whilst the immediate effects are of interest, the delayed effects (for example, 45–60 min post-exercise) are perhaps of greater importance because this is when the young people will be in their next academic lesson following a Physical Education lesson and/or break/recess, and where cognitive function will influence their learning. Interestingly, it has been suggested using a meta-analysis approach that similar effects are seen immediately following exercise and after a delay (Chang et al., 2012), but no study to date has examined both the immediate and delayed effects of exercise in young people in the same study.

Therefore, the aim of the present study was to test the hypothesis that a 10 min bout of repeated (~ 10 s) high intensity sprint-based running exercise would enhance cognitive function in an adolescent population, both immediately and following a delay. This novel study is important not only because high intensity intermittent activity is commonplace in young people, but also should beneficial effects be demonstrated, this could form the basis of future interventions aimed at increasing both physical activity levels and cognitive function/academic achievement in young people.

## 2 Methods

### 2.1 Participant characteristics

Forty-seven schoolchildren aged 12.6 (± 0.6) years were recruited to participate in the study. However, 3 participants failed to complete the study because they were absent from school for one of the experimental trials, thus 44 participants (21 male, 23 female) completed the study. During familiarisation, simple measures of height, body mass and waist circumference were taken. Height was measured using a Leicester Height Measure (Seca, Hamburg, Germany), accurate to 0.1 cm. Body mass was measured using a Seca 770 digital scale (Seca, Hamburg, Germany), accurate to 0.1 kg. These measures allowed the determination of Body Mass Index (BMI), calculated by dividing body mass [kg] by the square of the height [m^2^]. Waist circumference was measured at the narrowest point of the torso between the xiphoid process of the sternum and the iliac crest, to the nearest 0.1 cm. For descriptive purposes, the participant's anthropometric characteristics were (mean ± SD): height 154.9 ± 8.3 cm; body mass: 45.5 ± 9.0 kg; body mass index: 18.9 ± 3.2 kg/m^2^ (51.4 ± 29.3 percentile); waist circumference: 65.3 ± 7.5 cm.

### 2.2 Study design

The study was approved by the institution's ethical advisory committee. Participants were recruited from a local secondary school and in accordance with the ethical guidelines of the British Education Research Authority for school-based research, school-level consent was obtained from head teachers. In addition, written parental informed consent was obtained and a health screen questionnaire completed to ensure all participants were in good health. In addition, participants indicated their assent to take part in the study.

Each participant undertook a familiarisation session, which preceded the first of two experimental trials by seven days. During familiarisation, the protocol of the study was explained and participants were provided with an opportunity to familiarise themselves with the methods involved, which included completing the battery of cognitive function tests. In addition, participants were provided with an opportunity to ask questions and clarify any part of the tests they did not fully understand.

**Fig. 1:**
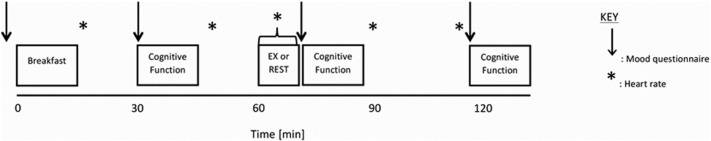
Experimental protocol.

The study employed a randomised crossover design, with participants blind until arrival at school on each day of testing. The experimental trials consisted of an exercise trial and a resting trial, thus participants acted as their own controls. During the exercise trial, participants completed 10 × 10 s sprints followed by 50 s active recovery 60 min following breakfast consumption. On the resting trial, participants remained seated in a classroom and continued to rest during this time. Trials were scheduled seven days apart and participants reported to school at the normal time. The experimental protocol is shown in Fig. 1.

### 2.3 Dietary control

Participants were asked to consume a meal of their choice the evening before their first experimental trial and repeated this meal for the subsequent trial. Following this meal, participants fasted from 10 pm. In order to maintain euhydration, participants were allowed to drink water ad libitum during this time. In addition, participants avoided any unusually vigorous exercise for 24 h prior to each experimental trial. Prior to each experimental trial a telephone call was made to participants to remind them of this information.

Following the overnight fast, participants reported to school at the normal time and completed the mood questionnaire. Due to the well documented effect of breakfast consumption (Cooper et al., 2011) and breakfast composition (Cooper et al., 2012a) on adolescents' cognitive function, participants were provided with a standardised breakfast, identical to that used in previous research (Cooper et al., 2012a). The breakfast consisted of cornflakes, milk, white bread (toasted) and margarine, and provided 1.5 g carbohydrate per kg body mass. As an example, the breakfast for a participant with a body mass of 50 kg consisted of 55 g cornflakes (Kelloggs, UK), 216 g 1% fat milk (Sainsbury's, UK), 42 g Kingsmill thick slice white bread (Kingsmill, UK) and 6 g margarine (Flora original, Flora, UK), providing a total energy content of 422 kcal and a macronutrient profile of 75 g carbohydrate (71% of energy intake), 14.3 g protein (14% of energy intake) and 7.2 g fat (15% of energy intake).

### 2.4 Exercise protocol

The exercise component of the study was completed in a school sports hall in groups of 8–12 participants. Prior to the main exercise protocol participants completed a standard warm-up, consisting of levels 1 and 2 of the multi-stage fitness test (Ramsbottom et al., 1988), followed by lower limb stretches.

Participants performed high-intensity intermittent running, consisting of 10 s sprints followed by 50 s active recovery (walking). This was repeated 10 times, thus participants performed 10 × 10 s sprints overall, and total exercise time was 10 min. Clear instructions were given to participants as follows:

*“The exercise you are going to be doing will involve 10 seconds sprinting followed by 50 seconds active recovery/walking. You will be moving up and down the 20 m shuttle you see in front of you. You clearly have a lane each – please make sure you stay in this lane at all times. The sprint you are going to be completing is a high intensity run. For health and safety reasons, please consider the need to turn at the end of each 20 metre shuttle and be particularly careful to stay in your lane as you turn. Please ensure that you run at a pace that you can maintain for all 10 sprints. This may mean that you run just below your maximum running pace.”*

Following these instructions, experimenters checked with participants to ensure understanding and there was an opportunity for participants to ask any questions regarding the exercise. At the end of every 10 s sprint, heart rate was recorded (Polar Wearlink heart rate monitor; Polar, Finland), and after sprint 5 and sprint 10, each participant's rating of perceived exertion was taken using the Robertson OMNI scale (Utter et al., 2002). Heart rate was also taken at several time points (15 min, 45 min, 90 min and 115 min) during both the exercise and resting trials.

### 2.5 Cognitive function tests

The battery of cognitive function tests consisted of the Stroop test, digit symbol substitution test and Corsi blocks test. The tests were administered via a laptop computer (Toshiba M750, Toshiba, Japan) and lasted approximately 10 min. Written instructions appeared on the screen at the start of each test, which were repeated verbally by an investigator. Each test was preceded by 3–6 practice stimuli, where feedback was provided regarding whether each participant's response was correct or not. Data from these practice stimuli were discarded and once the test started no feedback was provided. The cognitive function tests were administered in a classroom to groups of 8–12 participants at any one time, in silence and separated such that participants could not interact with each other during the cognitive testing. Participants were provided with sound cancelling headphones to minimise external disturbances. The same testing procedure has been previously used successfully in a similar study population (Cooper et al., 2011, Cooper et al., 2012a, Cooper et al., 2012b, Cooper et al., 2015) and the tests were administered in the order they are described here.

#### 2.5.1 Stroop test

The Stroop test measures the sensitivity to interference and the ability to suppress an automated response (Stroop, 1935). The Stroop test consisted of two levels. Both levels involved the test word being placed in the centre of the screen, with the target and distractor presented randomly on the right or left of the test word. The target position was counterbalanced for the left and right side within each test level. The participant was asked to respond as quickly as possible, using the left and right arrow keys, to identify the position of the target word.

The simple level contained 20 stimuli, where the test word was printed in white on the centre of the screen and the participant had to select the target word, from the target and distractor, which were also printed in white. The colour-interference (complex) level contained 40 stimuli and involved the participant selecting the colour the test word was written in, rather than the actual word (which was an incongruent colour), again using the right and left arrow keys to identify the target. The colour-interference level is a commonly used measure of selective attention and executive function (Leh et al., 2010, Miyake et al., 2000). The choices remained on the screen until the participant responded and the inter-stimulus interval was 1 s. The variables of interest were the response time of correct responses and the proportion of correct responses made.

#### 2.5.2 Digit symbol substitution test

The Digit Symbol Substitution Test (DSST) assesses general psychomotor speed and is well established in research, educational and clinical contexts. It consists of a list of digits which have to be matched with either the same digit (simple level), or a symbol which is to be paired to the digit via a key showing digit-symbol pairs (complex level). On both test levels, the variable of interest was the number of correct substitutions made in a 45 s period.

#### 2.5.3 Corsi blocks test

The Corsi blocks test is a measure of visuo-spatial working memory capacity that has been used for several decades (Corsi, 1972). A 3 × 3 grid of 9 blocks indicates a spatial sequence by changing the colour of one block at a time, and participants were then asked to repeat that sequence by clicking on the blocks in the same order. During the 12 trials, sequence length increased by 1 block location each time the participant correctly repeated the sequence. Any error in repeating a sequence led to the next sequence being shortened by one location. The variable of interest was mean memory span, calculated as the average length of the three longest correctly repeated sequences. This slight departure from the usual scoring procedure (Kessels et al., 2000) allows for more fine-grained scoring, as outcomes are fractions rather than just whole numbers.

### 2.6 Mood questionnaire

The mood questionnaire was a modified version of the ‘Activation-Deactivation Check List’ (AD ACL) short form, which has previously been shown as both a valid and reliable measure of mood (Thayer, 1986). The 20 item questionnaire was split into four components of mood; energy, tiredness, tension and calmness, each having five corresponding adjectives on the questionnaire. The original AD ACL short form was piloted in an adolescent population and subsequently five of the adjectives were changed to ensure suitability for the study population, with the modified version being previously used successfully in a similar study population (Cooper et al., 2011, Cooper et al., 2012a, Cooper et al., 2012b, Cooper et al., 2015). Participants were asked to respond to a series of adjectives regarding how they felt at that moment in time, on a scale of 1 to 5 (where 1: definitely do not feel, 3: unsure, 5: definitely feel). The scores on the adjectives for each component of mood were summed, providing an overall score for each component.

In addition, three visual analogue (VAS) scales were used to provide a measure of participants' hunger, fullness and concentration. The VAS scales consisted of a 10 cm line from one extreme to the other (i.e. not at all to very), with participants indicating the point on the line that applied to them at that moment in time.

### 2.7 Statistical analysis

Heart rate and mood data were analysed using SPSS (Version 22, SPSS Inc., Chicago, Il, USA) using a two-way (trial by time) repeated measures ANOVA, with paired comparisons conducted using paired samples t-tests. Cognitive function data were analysed using R (www.r-project.org). Response time analyses were performed using the nlme package for R, which implements mixed effect models and yields *t* statistics. Accuracy analyses were performed using the lme4 package for R, which also implements mixed effect models but with a binomial outcome data distribution and yields *z* statistics. Stroop test analyses were conducted using a three-way (trial by time by test level) interaction, followed by two-way (trial by time) interactions for each test level respectively, given that each test level requires different levels of cognitive processing (Miyake et al., 2000). The DSST and Corsi Blocks test were analysed using a two-way (trial by time) interaction. Where significant effects exist, effect sizes are presented as partial Eta squared values (η^2^_p_). Data are presented as mean ± standard deviation and for all analyses, significance was set as *p* < 0.05.

## 3 Results

### 3.1 Exercise and heart rate

Overall, heart rate during the exercise and recovery walks was 181 ± 13 beats·min^− 1^. Rating of perceived exertion, as assessed using the Robertson OMNI scale, was 4 ± 2 at the end of sprint 5 and 6 ± 2 at the end of sprint 10.

When assessed across the morning, there was significant trial by time interaction for heart rate (F_(3,81)_ = 1343, *p* < 0.0005, η^2^_p_ = 0.44, Table 2). Specifically, whilst heart rate was similar between trials at 15 and 45 min, heart rate was higher on the exercise trial at 90 and 115 min (15 and 40 min post-exercise respectively) (Table 1).

### 3.2 Mood

When assessing energy, tension and calmness using the ADACL there was no difference in the pattern of change across the exercise and resting trials (trial by time interactions, all *p* > 0.05). However, whilst tiredness was similar between the exercise and resting trials at 0, 30 and 110 min (0 min: resting 10 ± 4, exercise 10 ± 5, *p* = 0.739; 30 min: resting 9 ± 4, exercise 8 ± 4, *p* = 0.396; 110 min: resting 8 ± 4, exercise: 8 ± 4, *p* = 0.422), it was significantly higher immediately following the sprint-based exercise (at 75 min) when compared to the resting trial (resting 8 ± 4, exercise 12 ± 4, *p* < 0.0005). This led to a significant trial by time interaction (F_(3,93)_ = 10.5, *p* < 0.0005, η^2^_p_ = 0.25).

In addition, there was no difference in hunger, fullness or concentration (assessed using the VAS scales) across the morning between the exercise and resting trials (trial by time interactions, all *p* > 0.05).

### 3.3 Cognitive function tests

The data for each of the cognitive function tests at each time point across the exercise and resting trials can be found in Table 2.

### 3.4 Stroop test

#### 3.4.1 Response times

Response times were significantly quicker on the exercise trial when compared to the resting trial (resting: 946 ± 27 ms, exercise: 924 ± 25 ms; main effect of trial, t_(1,8455)_ = 3.2, *p* = 0.001, η^2^_p_ = 0.05). In addition, response times got significantly quicker as the morning progressed (time 1: 958 ± 24 ms, time 2: 926 ± 28 ms, time 3: 921 ± 27 ms; main effect of time, t_(1,8455)_ = − 5.7, *p* < 0.0005, η^2^_p_ = 0.11) and response times were significantly quicker on the simple level when compared to the complex level (simple: 796 ± 23 ms, complex: 1075 ± 29 ms; main effect of test level, t_(1,8455)_ = 46.5, *p* < 0.0005, η^2^_p_ = 0.86).

**Fig. 2:**
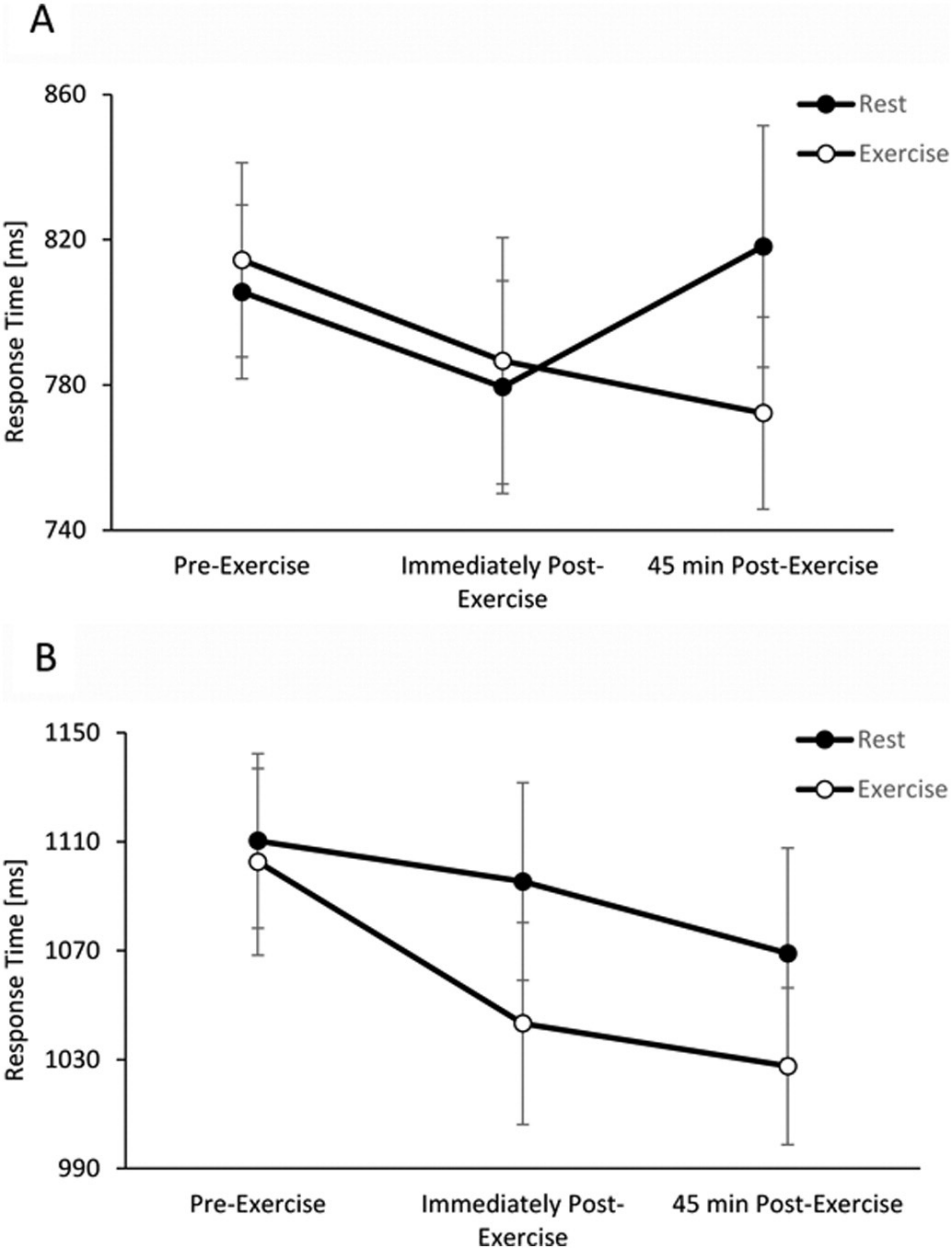
Response times across the exercise and resting trials on the simple (A) and complex (B) levels of the Stroop test. Data are mean ± SD.

There was no difference in the pattern of change in response times across the exercise and resting trials between the simple and complex levels of the Stroop test (trial by time by test level interaction, *p* = 0.562). However, when considering the simple and complex levels separately, some interesting effects were observed. On the simple level, whilst there was no immediate effect of exercise on response times, response times were significantly quicker 45 min post-exercise on the exercise trial compared to the resting trial (trial by time interaction, t_(1,3506)_ = 2.2, *p* = 0.027, η^2^_p_ = 0.03, Fig. 2a). However, on the complex level of the Stroop test, response times were significantly quicker immediately following sprint-based exercise when compared to the resting trial, whilst there was no difference in response times 45 min post-exercise (trial by time interaction, t_(1,4899)_ = 2.7, *p* = 0.038, η^2^_p_ = 0.02, Fig. 2b).

#### 3.4.2 Accuracy

There was no difference in accuracy between the exercise and resting trials on the Stroop test (main effect of trial, *p* = 0.703). However, participants become less accurate across the morning (time 1: 96.9 ± 0.4%, time 2: 94.8 ± 0.9%, time 3: 93.8 ± 1.0%, main effect of time, z_(1,8455)_ = 6.0, *p* = 0.004, η^2^ = 0.12) and were significantly more accurate on the simple level when compared to the complex level (simple: 96.5 ± 0.7%, complex: 93.8 ± 0.7%, main effect of test level, z_(1,8455)_ = 27.7, *p* < 0.0005, η^2^ = 0.39).

There was no difference in accuracy across the morning between the exercise and resting trials between the simple and complex levels of the Stroop test (trial by time by test level interaction, *p* = 0.401). Furthermore, when considering the simple and complex levels separately, there was no difference in accuracy across the morning between the exercise and resting trials on the Stroop test (trial by time interactions, simple: *p* = 0.194; complex: *p* = 0.970).

### 3.5 Digit symbol substitution test (DSST)

There was no difference in the number of correct substitutions made on the DSST between the exercise and resting trials (main effect of trial, *p* = 0.242), although the number of substitutions made increased across the morning (time 1: 24.5 ± 0.6, time 2: 25.4 ± 0.7, time 3: 25.8 ± 0.7, main effect of time, t_(1478)_ = 4.7, *p* < 0.0005, η^2^_p_ = 0.32). However, there was no difference in the number of correct substitutions made on the DSST across the morning between the exercise and resting trials (trial by time interaction, *p* = 0.689).

### 3.6 Corsi blocks test

There was no difference in the average span of correctly recalled locations on the Corsi blocks test between the exercise and resting trials (main effect of trial, *p* = 0.563). There was also no difference in the average span of correctly recalled items across the morning (main effect of time, *p* = 0.855). Furthermore, there was no difference in the average span of correctly recalled items on the Corsi blocks test across the morning between the exercise and resting trials (trial by time interaction, *p* = 0.289).

## 4 Discussion

The main finding of the present study was that sprint-based exercise enhanced the speed of executive function (as assessed by response times on the Stroop test) both immediately post-exercise and following a 45 min delay, while accuracy was maintained. However, there was no effect of sprint-based exercise on visuo-spatial working memory or general psychomotor speed (as assessed by the Corsi-blocks test and DSST respectively). The only component of mood to be affected by the sprint-based exercise was tiredness, which was significantly higher immediately following the exercise when compared to the resting trial. Therefore, the improvement in response times on the Stroop test immediately following exercise was evident despite a negative change in mood (higher self-report tiredness).

The present study is the first to examine the effects of sprint-based exercise on cognitive function in an adolescent population. The beneficial effects reported here are of particular interest given that the exercise model used more closely reflects the high-intensity, intermittent nature of physical activity patterns in young people (Bailey et al., 1995, Howe et al., 2010). These findings suggest that additional opportunities for high intensity physical activity incorporated into the school day could enhance executive function, and subsequently academic achievement (Duckworth and Seligman, 2005), in addition to the physical, emotional and social benefits of exercise which have been previously documented (American Academy of Pediatrics, 2013). This is a key consideration for school policy makers who often reduce the amount of time spent in Physical Education in favour of other ‘academic’ lessons (American Academy of Pediatrics, 2013).

The findings of the present study suggest that the effects of sprint-based exercise on cognitive function may be specific to the component of cognitive function examined. Specifically, whilst executive function was enhanced (as demonstrated by enhanced response times on the Stroop test with no concurrent decrease in accuracy), there was no effect on general information processing speed or visuo-spatial memory (as assessed by the DSST and Corsi blocks tapping test respectively). The beneficial effects on executive function are of particular interest given that executive function is responsible for more complex cognitive functions and decision making, which has been shown to be particularly important for reading ability (Savage et al., 2006) and academic achievement (Duckworth and Seligman, 2005) and are similar to the magnitude previously demonstrated following breakfast consumption and exercise (Cooper et al., 2015).

Taken together, the findings of the present study (examining sprint-based exercise) and those in the literature (examining moderate intensity exercise, e.g. Best (2010); Cooper et al. (2012b); Ellemberg and St-Louis-Deschênes (2010); Hillman et al. (2009); Stroth et al. (2009)) suggest that an acute bout of exercise enhances subsequent cognitive function in adolescents. However, the present study is the first to examine the effects of sprint-based exercise on adolescent's cognitive function, which may be different to those of more moderate intensity exercise, given the psychological, neuroendocrinological and metabolic mechanisms proposed to be affecting the exercise-cognition relationship (Audiffren, 2009, Dietrich, 2009, Kamijo et al., 2004, Kennedy and Newton, 1997, Lambourne and Tomporowski, 2010, McMorris, 2009). Specifically, the present study assessed the effect of high intensity sprint-based exercise on mood, with higher self-report tiredness seen immediately following exercise, with no changes in self-report energy, tension or calmness. Therefore, the improvement in executive function is seen despite a negative change in mood state, suggesting that mechanisms other than mood moderate the exercise-cognition relationship. That mechanisms other than mood moderate the exercise-cognition relationship is supported by the only study to directly compare moderate and high intensity on cognition, albeit in an adult population (Winter et al., 2007). Specifically, high intensity exercise was found to be more beneficial for cognitive function and it was suggested that this effect may be due to elevated BDNF and catecholamine concentrations (Winter et al., 2007).

Interestingly, the enhancement of response times on the Stroop test following high intensity sprint-based exercise is contrary to what would be expected given the suggestion of McMorris et al. (2016) that the stress of high intensity exercise (and the subsequent catecholamine response) has a negative effect on central executive tasks. However, McMorris et al. (2016) also suggest that the processing of sensory information by the parietal lobe, required for the completion of the Stroop test (Leung et al., 2000), is enhanced by the catecholamine response to high intensity exercise. Therefore, it could be that the positive effects on the processing of sensory information outweigh the negative effects on the central executive. However, these suggestions are based upon findings in animal models (Arnsten, 2011) and must be inferred cautiously in humans, where the effects warrant further investigation.

Another possible explanation of the domain specific effects seen in the present study, it is possible that whilst the Stroop test was sensitive enough to elucidate the beneficial effects of sprint-based exercise on adolescent's cognition, this was not the case for the DSST or Corsi blocks test. Upon inspection of Fig. 2, it is possible that the interaction effects seen on the simple level of the Stroop test are due to a slowing of response times 45 min post-exercise on the resting trial. However, on the colour-interference level there is a clearer enhancement of response times following the sprint-based exercise. This may suggest that more complex tasks show a greater beneficial effect of exercise, when compared to simpler tasks, in line with the effects of breakfast on cognition in young people (Cooper et al., 2012a, Cooper et al., 2012b). However, it is also possible that the effects of sprint-based exercise on cognitive function are domain specific, in line with previous reviews (Chang et al., 2012, Sibley and Etnier, 2003). Interestingly, a study by Budde et al. (2008) demonstrated that attention (as assessed by the d2 test) was enhanced following a maximal exercise test in young adults. However, the authors also suggested that this effect may be influenced by the habitual physical activity levels of the participants, with individuals who are more active demonstrating a greater exercise-induced improvement in attention. Future research is therefore required to examine both the domain-specific effects of high intensity exercise on cognition and the effect of baseline physical activity (and fitness) levels on this relationship.

Given the novelty of the present study it is difficult to draw firm conclusions in adolescents, but it would appear that a high intensity sprint-based exercise model has the potential to enhance executive function in this population. Interestingly, the beneficial effects were seen both immediately and 45 min post-exercise. Much of the literature to date has focussed only on the effects on cognitive function immediately post-exercise (Ellemberg and St-Louis-Deschênes, 2010, Stroth et al., 2009), but the present study has documented both the immediate and delayed effects. The findings of the present study are however in line with previous reviews in adults, suggesting that exercise enhances cognition both immediately post-exercise and following a delay. The time course of the exercise induced effects on cognition should continue to be examined in future studies, to allow schools and school policy makers to incorporate opportunities for physical activity, such as physically active breaks/recess in to the school day, to optimise learning.

A particular strength of the present study is the control of breakfast and the meal consumed the previous evening for each experimental trial, an advance on many previous studies that have not controlled diet. It is possible therefore that previous studies have their findings confounded by the well documented acute effects of nutrition on cognition in young people (Cooper et al., 2011, Cooper et al., 2012a, Hoyland et al., 2009). Furthermore, previous research suggests that breakfast and mid-morning exercise may interact to affect cognitive function in both adolescent (Cooper et al., 2015) and adult (Veasey et al., 2013) populations. Future research should continue to examine the combined acute effects of diet and exercise on cognitive function, including the interactive effects of nutrition with exercise of differing intensities and modalities.

Overall, the findings of the present study demonstrate that high intensity intermittent sprint-based exercise enhances executive function in young people, both immediately and 45 min post-exercise. Future research should continue to examine this relationship, including examining multiple components of cognitive function and the differential effects of exercise of differing intensities on young people's cognition, perhaps with the inclusion of a walking control group to allow direct comparison of differing exercise intensities. Furthermore, mechanistic measures such as BDNF, catecholamines or brain imaging techniques should be included in future research wherever possible, whilst also working within the ethical constraints of conducting research studies in young people.

## Conflict of interest

None declared.

## Figures and Tables

**Table 1:** Heart rate across the exercise and resting trials. Data are mean ± SD.

Time [min]	Heart rate [beats·min^− 1^]		*p* value
Exercise trial	Resting trial
15	92 ± 15	91 ± 17	0.476
45	91 ± 14	89 ± 18	0.982
90 (15 min post-exercise)	111 ± 14	90 ± 15	< 0.0005
115 (40 min post-exercise)	101 ± 14	90 ± 16	< 0.0005

**Table 2:** Cognitive function data across the exercise and resting trials. All data are mean ± SD.

			Resting trial			Exercise trial		
Test	Variable	Test level	Pre-exercise	Immediately post-exercise	45 min post-exercise	Pre-exercise	Immediately post-exercise	45 min post-exercise
Stroop	Response times [ms]	Simple	806 ± 24	779 ± 29	818 ± 33	814 ± 27	787 ± 34	772 ± 26
Complex	1110 ± 32	1095 ± 36	1069 ± 38	1103 ± 34	1043 ± 37	1028 ± 29
Accuracy [%]	Simple	97.9 ± 3.6	97.2 ± 4.1	95.0 ± 10.8	99.0 ± 2.9	94.8 ± 7.4	95.3 ± 6.1
Complex	94.9 ± 5.7	93.0 ± 6.4	92.2 ± 10.9	95.8 ± 5.2	94.1 ± 10.3	92.7 ± 7.7
DSST	Number of substitutions	Simple	25 ± 5	26 ± 5	27 ± 5	25 ± 4	26 ± 5	26 ± 5
Complex	24 ± 5	25 ± 5	25 ± 4	24 ± 4	25 ± 5	25 ± 5
Corsi blocks	Sequence length		5.5 ± 1.0	5.3 ± 0.9	5.4 ± 1.3	5.3 ± 1.1	5.5 ± 1.1	5.3 ± 1.0
